# Inferring Trust From Users’ Behaviours; Agents’ Predictability Positively Affects Trust, Task Performance and Cognitive Load in Human-Agent Real-Time Collaboration

**DOI:** 10.3389/frobt.2021.642201

**Published:** 2021-07-08

**Authors:** Sylvain Daronnat, Leif Azzopardi, Martin Halvey, Mateusz Dubiel

**Affiliations:** Department of Computer and Information Sciences, University of Strathclyde, Glasgow, United Kingdom

**Keywords:** HCI, AI, collaborative agents, user studies, performance, cognitive load, trust, reliability

## Abstract

Collaborative virtual agents help human operators to perform tasks in real-time. For this collaboration to be effective, human operators must appropriately trust the agent(s) they are interacting with. Multiple factors influence trust, such as the context of interaction, prior experiences with automated systems and the quality of the help offered by agents in terms of its transparency and performance. Most of the literature on trust in automation identified the performance of the agent as a key factor influencing trust. However, other work has shown that the behavior of the agent, type of the agent’s errors, and predictability of the agent’s actions can influence the likelihood of the user’s reliance on the agent and efficiency of tasks completion. Our work focuses on how agents’ predictability affects cognitive load, performance and users’ trust in a real-time human-agent collaborative task. We used an interactive aiming task where participants had to collaborate with different agents that varied in terms of their predictability and performance. This setup uses behavioral information (such as task performance and reliance on the agent) as well as standardized survey instruments to estimate participants’ reported trust in the agent, cognitive load and perception of task difficulty. Thirty participants took part in our lab-based study. Our results showed that agents with more predictable behaviors have a more positive impact on task performance, reliance and trust while reducing cognitive workload. In addition, we investigated the human-agent trust relationship by creating models that could predict participants’ trust ratings using interaction data. We found that we could reliably estimate participants’ reported trust in the agents using information related to performance, task difficulty and reliance. This study provides insights on behavioral factors that are the most meaningful to anticipate complacent or distrusting attitudes toward automation. With this work, we seek to pave the way for the development of trust-aware agents capable of responding more appropriately to users by being able to monitor components of the human-agent relationships that are the most salient for trust calibration.

## 1 Introduction

With advances in Artificial Intelligence, more and more intelligent agents are being deployed to aid human operators in completing tasks more effectively and efficiently (Chen et al., 2018). Human-Agent Collaboration (HAC) often requires users to validate or invalidate agents’ decisions in Command and Control scenarios, such as X-ray luggage screening (Chavaillaz et al., 2018; Merritt et al., 2013), collaborative bomb disposal robots (Costo and Molfino, 2004) or intensive care monitoring agents (Gholami et al., 2018). In these safety critical scenarios, collaborative agents facilitate the completion of tasks by aiding in the decision-making process (Emmerich et al., 2018).

Recently, there has been a renewed focus on developing intelligent collaborative agents able to work with human operators as teammates. In most situations where human-agent collaboration occurs, decisions need to be made in real-time, as interactions between agents and operators are continuous. For instance, rather than validating discrete actions made by an agent such as whether to give a patient insulin or not (Pak et al., 2012), users needs to actively work with agents to plan ahead and make decisions, such as monitoring and directing autonomous vehicles (Chen et al., 2015). Collaborative agents can be found in a multitude of contexts, some displaying anthropomorphic features (Pak et al., 2012; de Visser et al., 2016), such as voice or the likeness of a person, whereas some others provide help to human operators via textual or graphical interfaces (Mercado et al., 2015; Chen et al., 2018).

Many features influence the propensity of a human operator to trust and rely on an agent. Trust represents an important component of any scenario involving collaborative decision-making, as the perceived trustworthiness of an agent will dictate how a user will interact with it (Grodzinsky et al., 2011; Kunze et al., 2019; Kim and Lim, 2019; Tjøstheim et al., 2019). Past work has shown that an agent’s performance (in terms of reliability) as well as an agent’s behavior (in terms of predictability) are positively correlated with trust (Ogreten et al., 2010; de Visser et al., 2012). However, such studies have largely been conducted in turn-based settings (Pak et al., 2012; Correia et al., 2018) where operators and agents interact asynchronously. Human-agent teams often work together in real-time scenarios where the trust relationship evolves over time and is affected by various factors such as task performance and agents’ behaviors (Hoff and Bashir, 2015). Currently, there is a limited amount of work exploring the relationship between performance, predictability and trust when agents and humans work together in real-time collaborative settings. Since the focus of the current investigation is on different levels of agents’ performance and predictability and how it affects users, we decided to employ agents without any explicit anthropomorphic features (such as a human-like avatar or a voice).

### 1.1 Research Questions and Hypotheses

We ground this study in Human Factor research, which is based on the premise that the analysis of human-agent relationships can serve as means to understand users’ behaviors. In this work, we explore the relationship between users’ perceived trust and reliance on agents who exhibit different levels of predictability and reliability. Specifically, we attempt to address the following research questions. How, at the same level of agent’s reliability (performance), do changes in the agent’s predictability affect the following:

1) the users’ reliance on the agent?
2) the users’ workload when interacting with the agent?
3) the users’ perceived trust in the agent?

Previous work has shown that more reliable and more predictable agents tend to be trusted more by users in turn-based scenarios (Klein et al., 2004; Ogreten et al., 2010). Thus, we hypothesize that, at the same level of agent’s reliability (performance), agents exhibiting systematically biased behaviors (i.e., errors committed in a more predictable and consistent fashion) will be trusted more than agents exhibiting randomly varied behaviors (i.e., errors that are unpredictable and committed in an inconsistent way). We further hypothesize that it is possible to use behavioral data from human-agent interactions to model and infer users’ perceived trust in agents. The main contribution of our work lies in testing the impact of different degrees of agents’ reliability on the human-agent trust relationship in real-time scenarios. We use interaction data to model and determine how accurately can reliance, agents’ reliability and performance predict trust in automation.

## 2 Related Work

There has been a substantial amount of research on the measurement of trust in automation (see Schaefer et al. (2016) for a comprehensive review) which has typically been conducted using turn-based scenarios and survey instruments. Less attention, however, has been paid examining the effects of agent’s reliability and predictability in real-time human-agent collaborative tasks.

### 2.1 Trust in Automation

While there are many definitions of trust, we chose to use the one by Lee and See who define trust as: “the attitude that an agent will help achieve an individual’s goals in a situation characterized by uncertainty and vulnerability […] an agent can either be an automated system or another person that actively interacts with the environment on behalf of the person” (Lee and See, 2004, p.2). This definition is of particular relevance as it highlights that trust, as a concept, 1) does not differ between team members nor differentiate whether they are human or not, 2) involves collaboration and cooperation between team members, 3) is task dependent, and 4) evolves over time and through interactions. Trust is difficult to measure, monitor (Hoffman et al., 2013) and especially hard to assess in a real-time manner, as it is often too disruptive to interrupt and ask users to report trust ratings during the course of an interaction. Measuring and monitoring trust, however, is paramount to the success of human-agent interactions (Merritt et al., 2015). When trust in agents is too high, users tend to have a more complacent attitude, whereas when trust is too low, users tend to overlook or ignore agents’ inputs. Both complacency and distrust are undesirable as they negatively impact task performance (Singh et al., 1993a). Past work on the relationship between task performance and trust in automation indicated an “inversely proportional” relationship between trust in agents and cognitive load (Ahmad et al., 2019), as a decrease in trust levels is linked to an increase in cognitive workload.

In the context of human-agent interaction, inadequate trust in automated systems can be a factor leading to incidents, such as the ones related to the Boeing 737 Maneuvering Characteristics Augmentation System (MACS) (Sgobba, 2019). Through repeated interactions with agents, it has been shown that users’ trust evolves depending on the agent’s reliability (Merritt et al., 2015; Rossi et al., 2018). This process is called *trust calibration* (Freedy et al., 2007). As trust is a dynamic and task dependent concept, new methods are required to infer or predict a person’s trust in an agent, over time, given their interactions, rather than using post-hoc questionnaires to elicit trust. Knowing more about the process of trust calibration could in turn inform the design of future interactive systems (Jensen et al., 2020). Our paper aims to determine the impact of agents’ reliability and predictability on trust and performance via interaction data *and* questionnaires, and to investigate whether it is possible to use these information to predict trust.

### 2.2 Performance and Reliability

Performance is often considered as an outcome measure in cognitive tasks (Wiebe et al., 2014), while reliance often indicates the propensity of a user to take into account agents’ inputs in human-agent collaboration (HAC) scenarios. Past work has shown that an agent’s *reliability* and its task *performance* heavily influence users’ disposition to trust it (Robinette et al., 2017; Sheridan, 1989; Hoc et al., 2009). A comprehensive review by Honig and Oron-Gilad (2018) highlights past research focusing on agents’ failures and their impacts on users. In HAC scenarios, agents are generally introduced to reduce users’ cognitive workload, while trying to improve users’ situational awareness and overall task performance (Stowers et al., 2017; Demir et al., 2017; Karikawa et al., 2013; Fan et al., 2010).

Fan et al. (2008) tested different levels of agents’ variability (using systematic biases) in a turn-based Command and Control threat assessment task. They found that informing participants about the agent’s errors helps users to calibrate their trust accordingly, which leads to higher task performance. However, too much information regarding the agent’s errors can quickly overload users. In related work, Chavaillaz et al. (2018) investigated different levels of agents’ reliability on trust, reliance and overall task performance in a turn-based X-ray scanning scenario. Their results showed that, as agents reliability decreased, trust in the agents also decreased. Furthermore, they found that perceived reliability (i.e., how much a person is willing to rely on the agents’ inputs) is also affected by the capabilities of the automated system. In their studies, users’ perception of the reliability of agents was more accurate when interacting with low performing agents, compared to high performing ones. In addition to the studies focusing on different degrees of reliability, the work of Shirado and Christakis (2017) explored turn-based coordination problems and found that error-prone agents (up to 30% loss in accuracy) could be beneficial to collaborative performance as it reduces the probability of the user being complacent while interacting with the agent. Similarly, the work of Salem et al. (2015), found that the type of error an agent is making (breach of privacy, violations) has an impact on the way users perceive the agent, and will affect how much users are willing to rely on it in subsequent interactions. Given the evidence of past research, it is clear that the performance of an agent (its reliability) as well as the agent’s behavior (its predictability) impact trust.

### 2.3 Real-Time VS Turn-Based Tasks

As previously mentioned, most studies in the area of trust in agents have been performed using turn-based scenarios, where the agent provides options that users either accept or reject (Fan et al., 2008; Chavaillaz et al., 2018; Shirado and Christakis, 2017). These scenarios usually offer users more time to assess a situation and react accordingly. However, agents are being integrated in more complex environments where decisions need to be made in real-time. Contrary to a turn-based activity where the human operator can afford to wait and get the full information about a situation before making decisions, real-time collaborative scenarios involve continuous monitoring and decision making in order to anticipate future actions and plan alternatives (Newn et al., 2017). These collaborative real-time situations differ from turn-based tasks, in which users can afford to analyze information and plan adequate action. It is then increasingly important to study the dynamics of trust relationships in real-time scenarios and to investigate whether trust can be predicted from past interactions. In this paper, we focus on exploring how agents’ reliability and predictability influence users’ trust, reliance and cognitive workload as well as the resulting impact on task performance in a real-time human-agent collaborative scenario.

## 3 Method

To answer our research questions and test our hypotheses, we designed a 2 × 2 within groups study employing a repeated measures design. Participants undertook a Command and Control task that involved agents having different levels of reliability (low and high) affecting how well the agents performed at the task, and predictability (systematically biased or randomly varied when targeting) which affected how predictable the behavior of the agents was. We also added two baseline conditions, one in which users played without any agent and another where the agent was flawless (highly reliable). The experiment was undertaken in the context of a collaborative missile command scenario where participants and agents need to work together to defend cities from incoming enemy missiles. Ethics approval for this study was obtained from the University of Strathclyde’s Department of Computer and Information Sciences (Approval No. 793).

### 3.1 Missile Command Scenario

**FIGURE 1:**
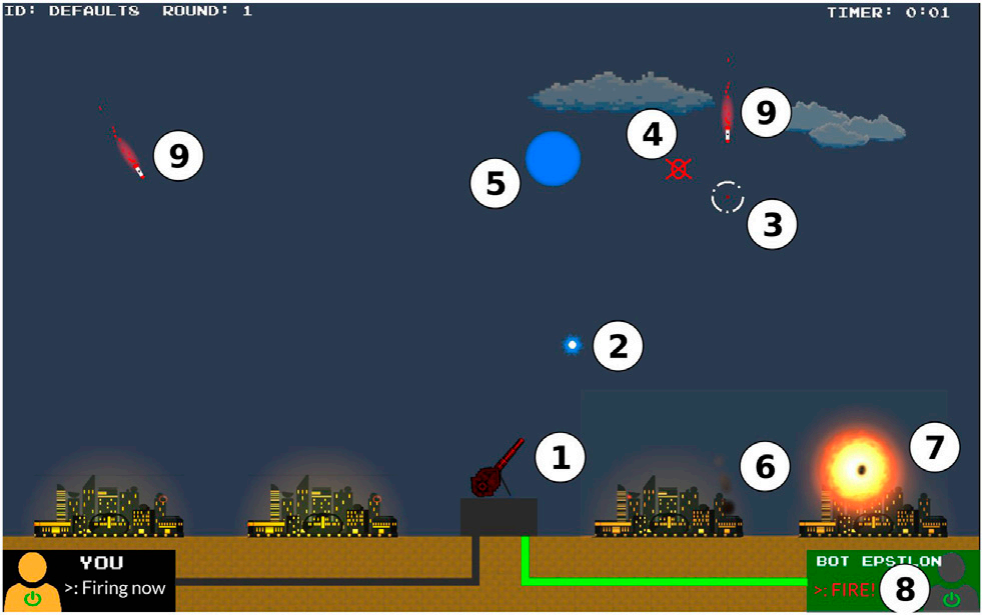
Annotated screen-capture of the missile command scenario.

Our real-time interactive task consists of aiming at and destroying missiles appearing from the top of the screen in order to protect cities positioned at the bottom of the screen. To do so, participants can freely move a crosshair across the screen and fire projectiles in the direction of their choice. In most of the scenarios we designed, participants can collaborate with agents capable of aiding with the aiming process. At any moment, however, participants can choose to override the agents’ inputs and manually move the crosshair. In all scenarios, only participants can fire projectiles to destroy incoming missiles (this design decision was taken to lessen the likelihood of users’ complacent behavior). Game-based frameworks are often used to study human-agent interactions due to their immersive and easy-to-access nature (Almajdalawi et al., 2016; Wang et al., 2015). Similarly to previous studies on trust (Correia et al., 2018; Sordoni et al., 2010), this scenario provides a controlled environment where human-agents interactions can be monitored and recorded. Figure 1 shows the overview of our interactive scenario, where the main elements are numbered and described as follows:

1) **Gun-turret**: controlled by either the participant or the agent in order to aim and target incoming missiles. All of the projectiles are fired from the turret.
2) **Projectile**: fired by the participant, it travels at a fixed speed until it explodes in a small circular area. If a missile lies within this area, it is destroyed.
3) **Crosshair**: provides a visual indication of where the participant or agent is aiming. The crosshair changes its color depending on who is controlling it (yellow for the participant, white for the agent, and dark-grey for neither.)
4) **Red Indicator Area**: appears when a projectile is fired to show participants the area where the projectile will explode.
5) **Projectile’s explosion (halo)**: In order to get destroyed, the missiles have to enter within the radius of such explosion.
6) **City**: Assets that the participants are tasked to protect.
7) **Missile Impact**: when a missile reaches a city, it produces an orange/red explosion with smoke emanating from the city.
8) **User and Agent panels**: The user’s panel (on the bottom left of the screen) and the agent’s panel (on the bottom right) light up in green when one of them is moving the crosshair.
9) **Enemy missile**: travels at a fixed speed and angle depending on the task difficulty. At the end of a session (with or without an agent), participants are shown how many missiles they hit and/or missed. All missiles missed will eventually hit a city.

### 3.2 Agents: Reliability and Predictability

**FIGURE 2:**
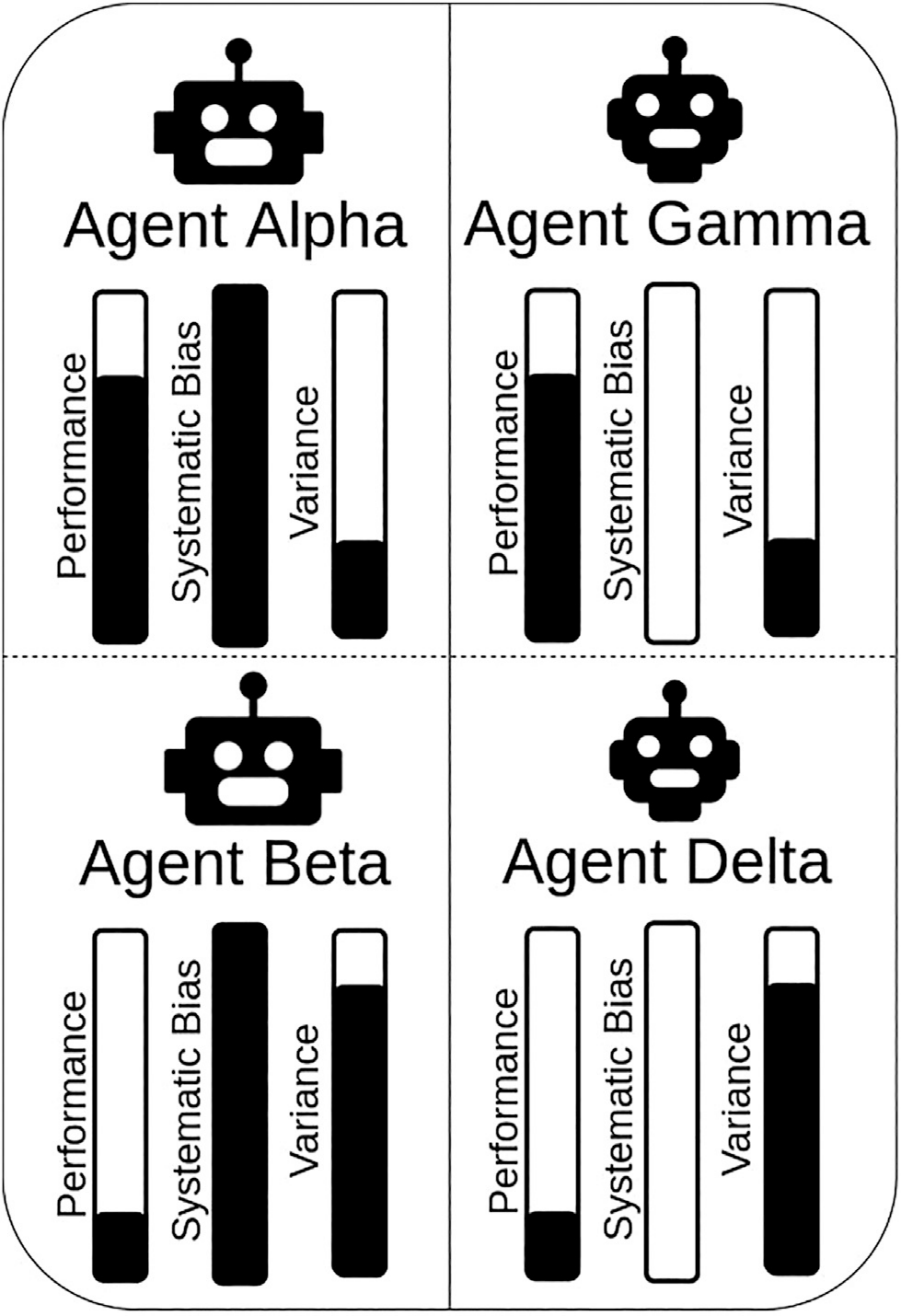
Agents with different degrees of predictability (behaviors) and reliability (performance) were created for this study. Systematic bias and random variance were used to respectively constrain how predictable and accurate the agents’ accuracy was.

Participants interacted with five different agents. Each of the agents varied in its ‘targeting style’, which was controlled to create different levels of performance and predictability. Agent names were introduced to make it easier for participants to refer to any particular agent. Agents Alpha and Beta were designed to be more predictable with respectively a high (Alpha) and low (Beta) level of performance. Agents Gamma and Delta were designed to be less predictable with respectively a high (Gamma) and low (Delta) level of performance. Figure 2 shows the different combinations of agents used, which we refer to as: Alpha, Beta, Gamma, and Delta (A,B,C,D).

**FIGURE 3:**
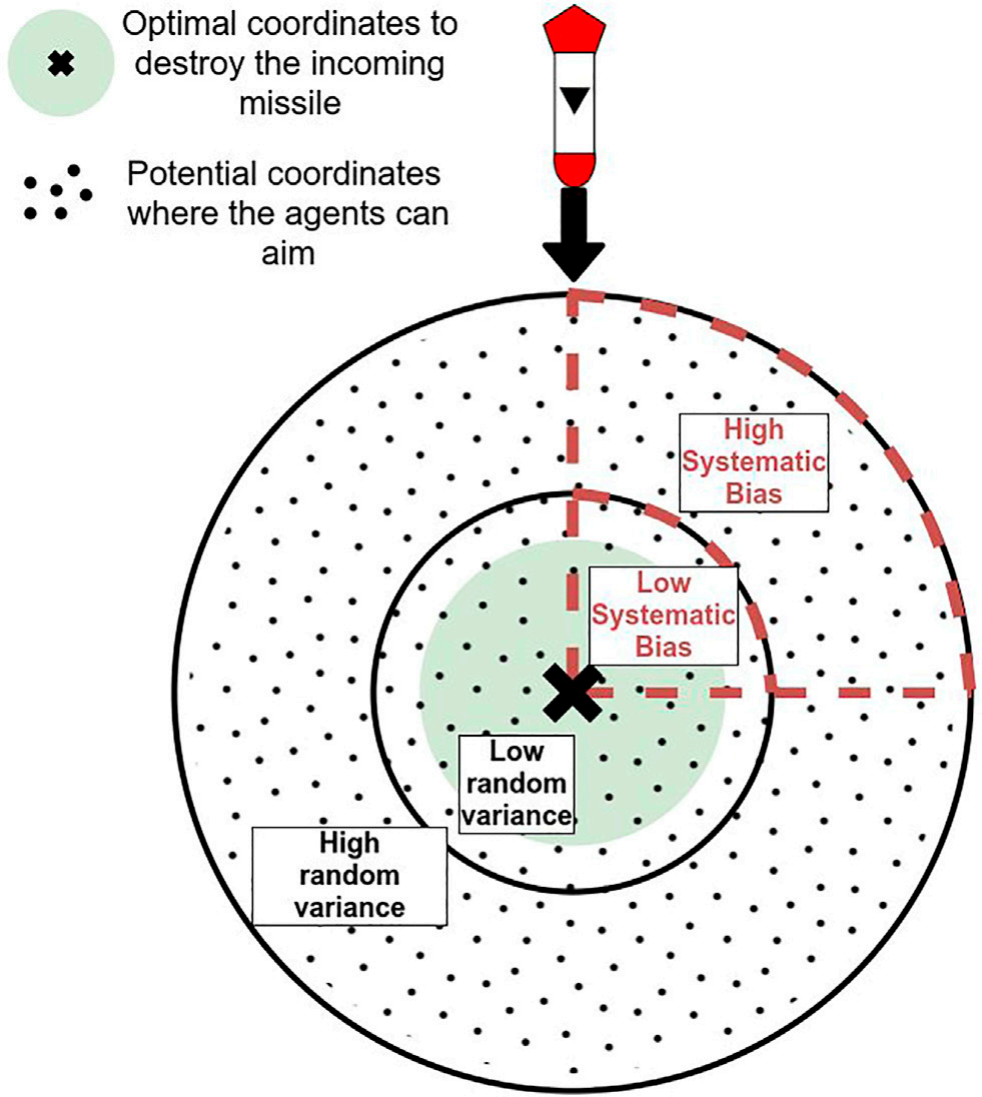
Visualization of the different biases applied to the agents in the study (not to scale). The greater the bias, the lower the accuracy of the agent. For the systematic bias, a “quadrant” is randomly chosen for each participant at the beginning of a session. Low systematic bias and low random variance or high systematic bias and high random variance result in the same performance output.

All agents had a certain degree of variance in their aiming’s accuracy such that, for a given target, a certain degree of error would be applied to the targeting. This variance in the agent’s performance was calculated using a random Gaussian distribution with a fixed *σ* for each level of agent’s performance. The greater the variance (and thus the *σ*), the less accurate the agent’s aim, leading to worse task performance (see Figure 3). In addition to variance, agents Alpha and Beta had their aiming systematically biased in a particular direction: 1) always above and to the right of their target, 2) always below and to the left, 3) always above and to the left, 4) always below and to the right. The direction of the systematic bias was randomly selected at the beginning of the experiment, per participant, and kept constant during the condition. By randomly selecting the direction of the bias, we ensured that our findings were not constrained by a specific type of systematic bias. This systematic bias impacted the agents’ targeting behaviors, but not their performance, which were only impacted by random variance.

Agents’ performance was calibrated using simulations where the agents completed the task by themselves (e.g., the same task without users). In these simulations, we calculated the agent’s performance based on Recall scores described in Section 3.5. We then ensured that the performance of agents with a similar level of performance was not significantly different using t-tests, in order to ensure that high or low degrees of predictability would not impact agents’ performance, thus allowing comparisons. While comparing the Recall scores of agents Alpha and Gamma (low performing agents), a *t*-test yielded p>0.05. Similarly, t-tests performed using Recall scores of agents Beta and Delta also yielded p>0.05. Agents Beta and Delta were tuned to have high performance (approx. 0.7 Recall scores or 70% of the targets being hit), while agents Alpha and Gamma were tuned to have low performance (approx. 0.3 Recall scores, or 30% of the target beings hit).

By controlling agents’ performance and predictability, we were able to test our main hypothesis using a 2 × 2 design. In addition to the aforementioned agents, we also included a perfect agent: Epsilon which exhibited no bias and no variance–and thus had the highest reliability and predictability out of all of the agents (effectively serving as an upper bound on performance).

### 3.3 Rounds & Difficulty

During each interaction with an agent, participants went through three rounds which lasted for 90 s each. This duration was set so that participants had enough time to familiarize themselves and adapt to the agents, while ensuring that the experiment could be completed within an hour (lessening participants’ fatigue). Each round increased in difficulty (going through “Easy”, “Medium” and “Hard” difficulty levels). In the “Easy” level, missiles spawned every 4 s at a speed of 100 pixels per second, for the “Medium” difficulty level, missiles spawned every 2 s with a speed of 150 pixels per second, and finally, for the “Hard” difficulty level, missiles spawned every second with a speed of 200 pixels per second. These settings were calibrated during pilot testing with ten participants, to make sure that changes in difficulty were noticeable without completely overwhelming participants (see Section 3.4 for a more detailed description of the pilot study).

### 3.4 Piloting

Before conducting the main study, a formal pilot experiment was carried out. Ten participants were recruited from our local Computer Science department. This pilot experiment focused on calibrating the single player (no agent) experience, as well as core gameplay elements such as the controls, visuals and overall difficulty of the game.

To evaluate participants’ performance, we used F1 scores. F1 is a metric related to participants’ overall task performance and is computed using the number of missiles participants hit, the number of shots fired and the total number of missiles present in each level. Fore more information, all of the performance metrics are detailed in Section 3.5. F1 scores varied between 0.88 for the “Easy”, 0.77 for the “Medium” and 0.46 for the “Hard” difficulty levels. We then decided to increase the speed of missiles in the “Medium” difficulty level to intensify its complexity.

Since the radius of the projectile’s explosion was reported to be too wide during the post-hoc interview, we decided to reduce it from 60 to 45 pixels. The speed at which participants were able to move the cross-hair was perceived to be too slow, therefore we decided to increase it from 600 to 800 pixels per second. During further informal pilots, participants gave additional feedback regarding how distinguishable colors used to indicate whether the user or the agent was in control were. Based on the received feedback, we chose to associate the agent control with yellow and the user control with white.

### 3.5 Interactions and Performance Logging

Participants’ interactions were logged during each task. Logging included the number of shots fired, missiles destroyed, missiles on screen, time spent controlling the crosshair by the user (in seconds) and the distance that the crosshair traveled while moved by the user. The above metrics where logged for all scenarios, both with and without agents. Using the data collected during these interactions, we then calculated the following task performance measures:

$$\mathrm{Precision}=\frac{\#MissilesDestroyed,}{\#ShotsFired}$$ (1)

$$\mathrm{Recall}=\frac{\#MissilesDestroyed,}{\#IncomingMissiles}$$ (2)

$$F1=2*\frac{Precision*Recall}{Precision+Recall.}$$ (3)

Higher precision indicates greater accuracy (fewer attempts to hit a target), while higher recall indicates greater task performance (less damage being sustained by the cities). F1 is the harmonic mean of precision and recall which provides a combined measure of performance. The user control time was computed as the number of seconds when participants were controlling the crosshair during each round (a greater user control time indicates less reliance on the agent).

### 3.6 Questionnaires

Participants completed a NASA TLX questionnaire, which consists of six individual rating scales that are commonly used to measure cognitive workload (Hart, 2006). In our study, we report RAW TLX (Cao et al., 2009) scores. To measure trust in the agents, we used a single statement at the end of each round: “I can trust the agent” graded on a 11-points Likert scales ranging from 1 (complete distrust in the agent) to 11 (total trust in the agent). The scale was adapted from the work of Jian et al. (2000).

### 3.7 Dependant and Independent Variables

The independent variables in this study are as follows:

• **Agent Behaviors**: Predictability (high or low) and Reliability (high or low).
• **Difficulty** per round (Easy, Medium, and Hard).

The dependent variables in this study are as follows:

• **Time in Control**: The time participants and agents spent controlling the crosshair for each round.
• The number of **missiles destroyed**, the number of **projectiles fired** and the total number of **hits sustained by cities**, per round. These metrics are used to assess task performance in the form of Recall, Precision and F1 scores (see Section 3.5).
• **Distance traveled by the crosshair** when the user or an agent were in control of it.
• **NASA TLX** (NASA, 1986) ratings scales employed to measure participants’ cognitive workload after each round of the game.
• **Single Trust Question** (Jian et al., 2000; Entin and Serfaty, 2017) provided at the end of each round. Higher ratings indicate higher reported trust.

### 3.8 Experimental Procedure

Participants were briefed on the experiment and asked to provide consent required to undertake the study. After completing a demographic questionnaire, participants were first given a short tutorial on how to play the game and interact with the agents. They were instructed that their goal was to work with the agents to protect cities by destroying all incoming missiles. They were informed that they could always correct the agents’ aiming if they desired to do so. Following this briefing, participants completed a session without the assistance of an agent. The purpose of this session was to record individual users’ performance. Participants then played with all of the other agents. The sequence in which participants interacted with each agent was randomized using a William Square design in order to mitigate possible learning effects (Williams, 1949). During each session, participants worked through three rounds of low to high levels of difficulty. At the end of each round, participants were asked to rate their trust in the agents. At the end of each session, participants were asked to complete the NASA Task Load Index (TLX) questionnaire. At the end of the study, which lasted for approximately an hour, participants were compensated for their time with a shopping voucher worth *£*10.

### 3.9 Demographics

**FIGURE 4:**
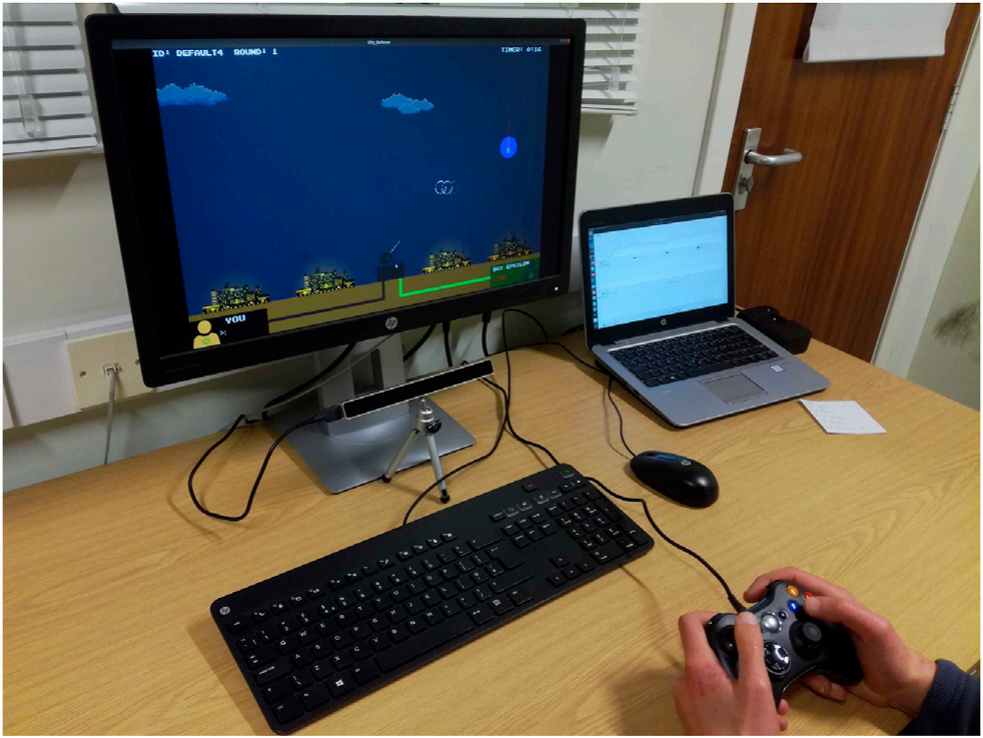
Picture of the experimental setup used during the study. Participants took part in the experiment in a quiet lab, using a Xbox360 controller and playing on a 24″ 1920 × 1,080 monitor.

Participants were recruited through mailing lists and flyers posted on our university campus. Figure 4 presents a picture of our experimental apparatus. We recruited a total of 30 participants (14M,16F) with ages ranging from 19 to 38 years old (M=27±5.19). Most participants were enrolled as postgraduate students. Ratings from the Complacency Potential Rating Scale (CPRS) (Singh et al., 1993b) were used to evaluate general attitude toward automation. CPRS scores ranged from 55.57 to 90.84 (M=72.55±9.3) which indicates that our sample consisted of participants who were more likely to rely on automation than not (Singh et al., 1993b). Overall, the distribution of scores was homogeneous enough that our sample could not be divided in different group representing distinct attitudes toward automation.

## 4 Results

In this section, we present results regarding task performance, users’ reliance on agents, workload and reported trust in the agents. Then, we model and predict trust ratings using aforementioned performance and user behavior metrics. To compare our different conditions, we first used repeated measures ANOVAs (for which we are always reporting *p* and *F* values) and then performed follow-up pairwise comparisons using T-tests, if statistically significant results were found (p<0.05). Bonferroni corrections were applied to determine which conditions were significantly different. For T-tests, we always report *p*-values as well as the effect size using Cohen’s *d* values (Note that 0.5<d<0.8 is considered a medium effect size, whereas d>0.8 is a large effect size (Cohen, 1988)). In Tables 1 and 2, if the score in a given condition was significantly better than in other conditions, we denote it by using superscripts letters (N for no agent and A, B, G, D, E for each agent).

**TABLE 1:** Metrics related to performance (Recall, Precision and F1, higher = better) and reliance (User control time (in seconds) higher = less reliance on the agent). Superscript letters next to the results indicate which agents yielded significantly worse scores (p<0.05).

	No agent	Agent alpha rel+/pred+	Agent betarel-/pred+	Agent gamma rel+/pred-	Agent deltarel-/pred-	Agent epsilon highest reliability
**Recall**	0.64± 0.03^*D*^	0.82±0.02^*NDBG*^	0.60±0.03	0.72±0.02^*NDB*^	0.58±0.03	0.98±0.01^*NDBAG*^
**Precision**	0.57±0.02^*DB*^	0.60±0.02^*DBG*^	0.50±0.03	0.53± 0.02^*D*^	0.47 0.02	0.86±0.01^*NDBAG*^
**F1**	0.60±0.02^*DB*^	0.68±0.02^*NDBG*^	0.54±0.03	0.60±0.02^*DB*^	0.51±0.03	0.91±0.01^*NDBAG*^
**User ctrl time**	25.12±0.96^*EAG*^	5.34±0.83^*E*^	24.18±1.16^*EAG*^	10.61±1.09^*EA*^	27.68±1.29^*BEAG*^	1.02±0.43

**TABLE 2:** Metrics related to cognitive load and trust ratings. Superscript letters next to the results indicate which agents yielded significantly worse scores (p<0.05).

	Agent alpha rel+/pred+	Agent beta rel-/pred+	Agent gamma rel+/pred-	Agent delta rel-/pred-	Agent epsilon highest reliability
**Raw TLX**	9.64±0.34^*E*^	14.62±0.38^*EAG*^	11.57±0.30^*EA*^	15.47±0.31^*BEAG*^	4.79±0.36
**Trust ratings**	7.82±0.26^*DBG*^	2.16±0.16	6.28±0.28^*DB*^	2.17±0.16	10.61±0.13^*DBAG*^

A main effect analysis was conducted to test the impact of agents’ predictability and reliability on participants using an univariate linear regression. We found that participants interacting with high predictability agents (Alpha and Beta) performed better in terms of Recall scores (p<0.0001, F=237.8), trusted the agents more (p<0.0001, F=139.3), relied on the agents more (p<0.0001, F=220.3) and reported lower cognitive load (p<0.0001, F=370.8). Similarly, participants that interacted with high reliability agents (agents Alpha, Gamma and Epsilon) performed better in terms of Recall scores (p<0.0001, F=175.1), trusted the agents more (p<0.0001, F=28.37), relied on the agents more (p<0.0001, F=829.1) and reported lower cognitive load (p<0.0001, F=609.4). The following subsections highlight comparisons and results related to all of our main dependent variables.

### 4.1 Performance

**FIGURE 5:**
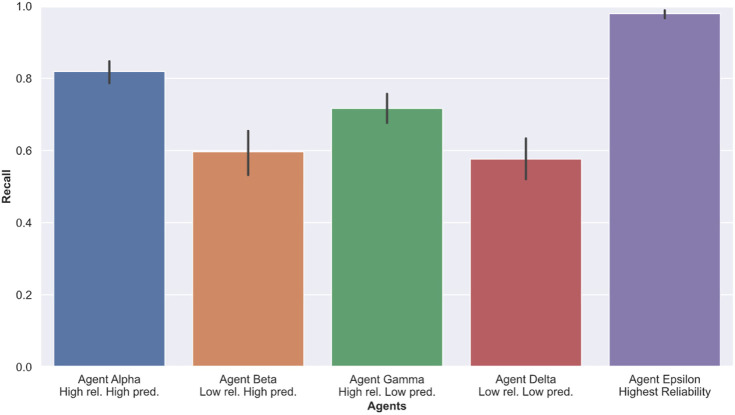
Average Recall scores for each session with agents. A higher score indicates better performance.

**FIGURE 6:**
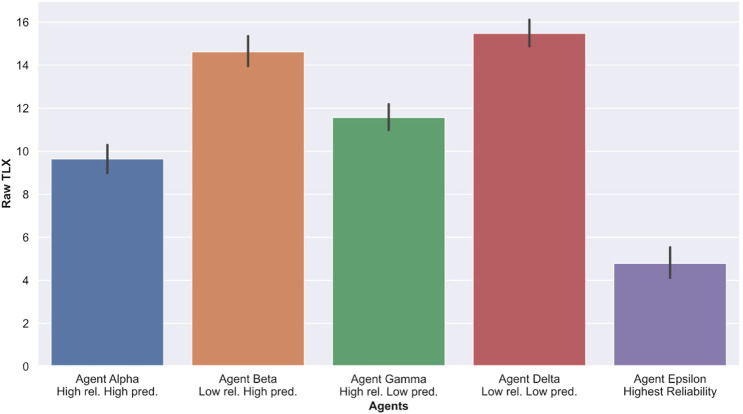
Relationship between participants’ Recall and Precision scores for each session (with or without agents). Each dot represents one participant.

To measure task performance, We computed Recall, Precision and F1 scores based on the number of shots fired, missiles hit and total missile present in each level of our experiment. Recall, Precision and F1 scores are detailed in Section 3.5. Table 1 and Figure 5 show the average task performance achieved by participants in each condition. These scores are averaged over all three levels of difficulty. Figure 6 shows the relationship between Recall and Precision scores for all participants and session. From consulting Figure 6, we can see that participants achieved better Recall scores while interacting with high reliability agents (Alpha and Gamma) than on their own (without an agent). Participants performing poorly in the no agent session benefited the most from this increase in performance. As expected, participants performed the best with agent Epsilon (highest reliability) compared to any of the other conditions across all measures. When interacting with Alpha (high reliability, high predictability) and Gamma (high reliability, low predictability), participants were able to achieve higher precision scores than by themselves (no agent), but performed worse with Beta (low reliability, high predictability) and Delta (low reliability, low predictability) across all performance measures, yielding lower Recall, Precision and F1 scores. ANOVA testing yielded significant results for Precision scores (p<0.0001, F=3.55), Recall scores (p<0.0001, F=37.47), and F1 scores (p=0.0002, F=9.65). Follow-up pairwise comparisons revealed statistically significant results between Alpha (high reliability, high predictability) and Gamma (high reliability, low predictability) for Precision (p=0.0001, d=0.54), Recall (p<0.0001, d=0.86) and F1 (p<0.0001, d=0.66) scores.

### 4.2 Reliance

**FIGURE 7:**
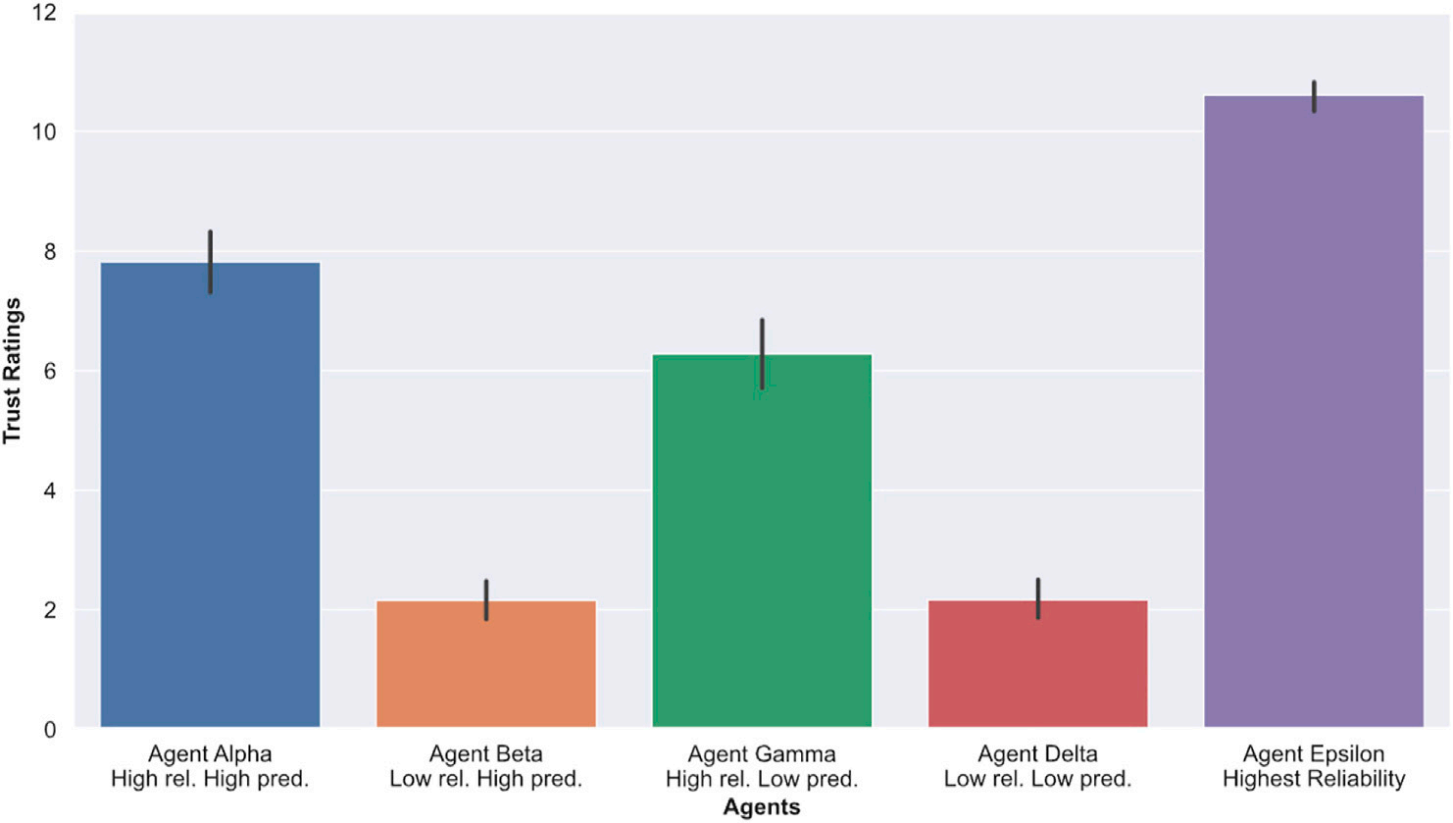
Average amount of time spent by each participant to correct the agents. A higher amount of timeindicate less reliance on the agents.

To measure how much participants relied on an agent, we computed the duration for which each participant controlled the crosshair. Participants controlling the crosshair for a longer period of time suggests that they relied on the agents less (and vice versa). Table 1 and Figure 7 show the average amount of time (in seconds) participants spent in control of the crosshair (denoted as User Ctrl Time). As expected, we observed that participants spent less time controlling the crosshair when working with Epsilon (highest reliability) compared to any of the other conditions, with or without agents. In addition, participants spent significantly more time controlling the crosshair (p<0.0001) when collaborating with low reliability agents (Beta and Delta) compared to high performance agents (Alpha and Gamma). ANOVA testing yielded statistically significant results (p<0.0001, F=22.70) when comparing the overall user control time, however follow up pair-wize comparisons indicated that these differences were only significant between Alpha (high reliability, high predictability) and Gamma (high reliability, low predictability) with p<0.0001 and a large effect size (d=0.81).

### 4.3 Cognitive Load

**FIGURE 8:**
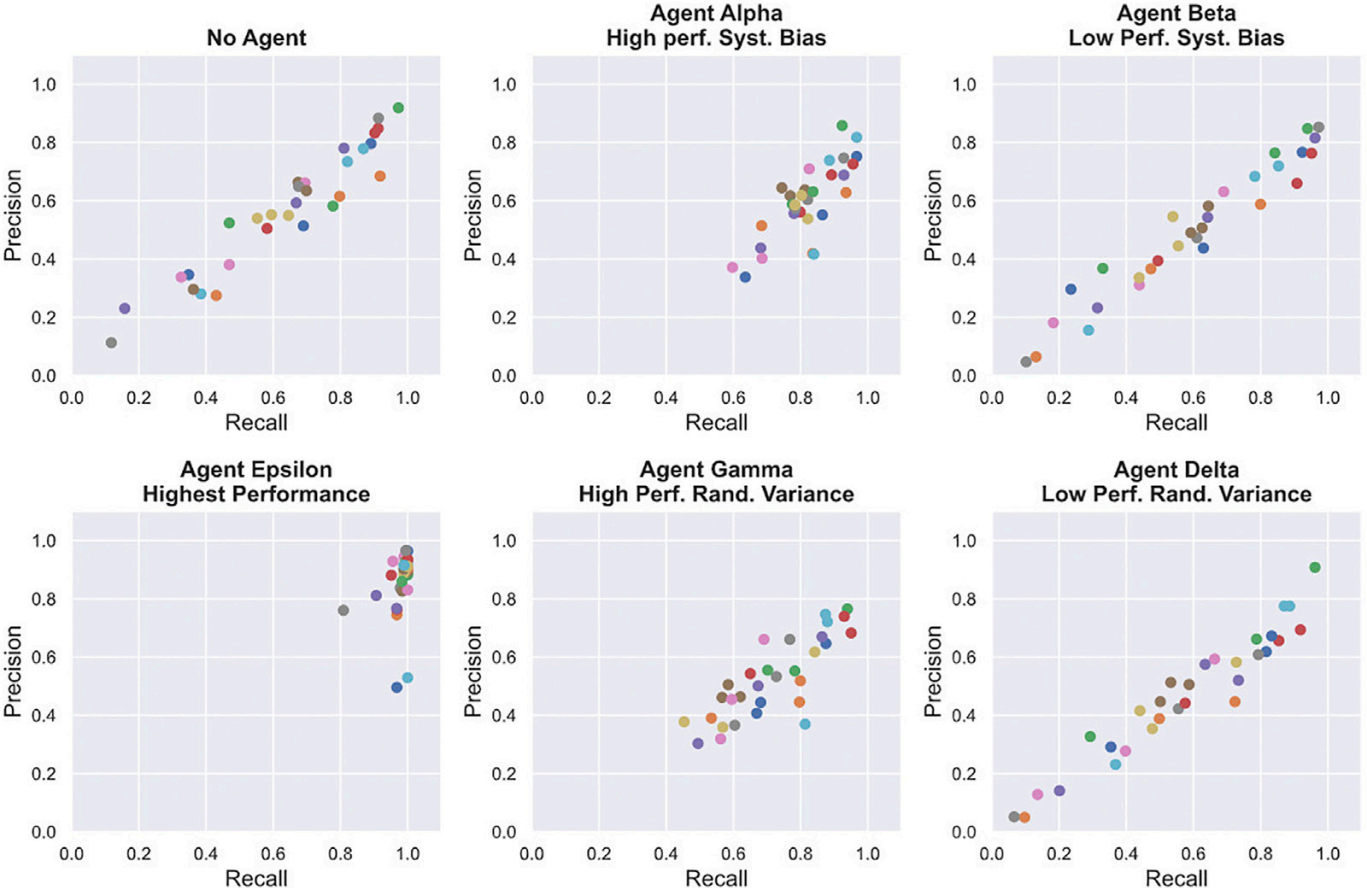
Average Raw NASA TLX ratings for each sessions with agents. Higher scores indicate greater cognitive loads.

To measure Cognitive load, we used the Nasa TLX survey instrument detailed in Section 3.7. Higher scores indicate a greater reported workload. As presented in Table 2 and Figure 8, we observe that participants reported much lower cognitive load (NASA TLX scores) when interacting with agent Epsilon (highest reliability) compared to any of the other agents. Furthermore, participants reported much higher cognitive load when interacting with low reliability agents (Beta and Delta) compared to high reliability ones (Alpha and Gamma). When comparing overall Raw Nasa TLX scores, an ANOVA yielded significant results (F=8.73, p=0.006). While performing pairwise comparisons, we found that participants perceived the high reliability, high predictability agent (Alpha) as significantly less cognitively taxing than the high reliability, low predictability agent (Gamma) with p=0.0061, d=0.623. In addition, participants found the agent with low reliability and low predictability (Delta) as significantly more cognitively taxing than the agent with low reliability and high predictability (Beta) with p=0.0473, d=0.26.

### 4.4 Trust

**FIGURE 9:**
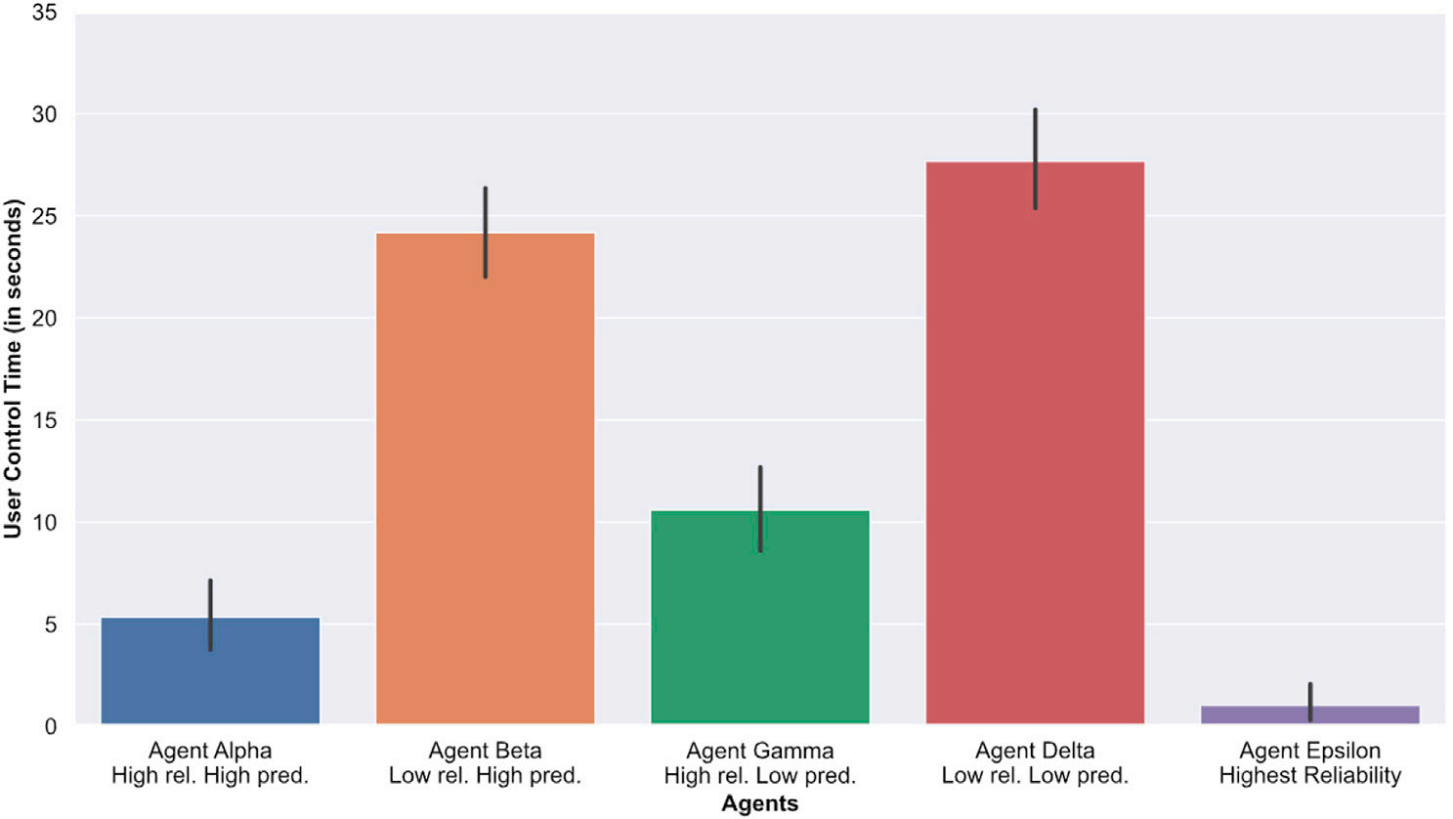
Average reported trust in the agents. Higher scores indicate greater trust in the agents.

To measure trust, we asked participants to rate their perceived trust in the agent on a single-element trust rating scale marked from 1 to 11, with a lower score indicating a lower reported trust in the agent. Table 2 and Figure 9 indicate that, on average, participants trusted agent Epsilon (highest reliability) more than any of the other agents, which was expected. In addition, trust ratings of agents with low reliability (Beta and Delta) were on average much lower than agents with high reliability (Alpha and Gamma). When comparing answers pertaining to the trustworthiness of agents, an ANOVA yielded significant results (F=7.80, p=0.0018). While performing pairwise comparisons, we found that participants rated Alpha (high reliability, high predictability) significantly higher than Gamma (high reliability, low predictability) with p=0.0011, d=0.86. Overall, no significant results were found when comparing Beta (low reliability, high predictability) to Delta (low reliability, low predictability). These results indicate that, at the same high level of agents’ performance (high reliability), participants were more trustful of an agent with high predictability (Alpha) than an agent with low predictability (Gamma).

### 4.5 Predicting Trust

To examine how different variables influenced trust, we analyzed correlations between trust ratings, task difficulty, the reliance metric (user control time), cognitive workload (NASA TLX scores) and performance metrics (Precision, Recall and F1 scores). Table 3 reports information regarding Spearmans’ *ρ* and *p*-values of each correlation tests. From Table 3, we can see that participants’ reliance on the agents (as measured by user control time) led to the highest correlation (ρ=−0.801, p<0.001) followed by Cognitive Load (Raw TLX scores) with ρ=−0.730, p<0.001, whereas performance metrics (Recall, F1 and Precision) resulted in lower correlations ranging from 0.50 to 0.61.

**TABLE 3:** Spearmans correlation tests between participants’ behavioral metrics (performance and reliance) and reported trust ratings. A higher *ρ* scores indicates greater correlation.

**Parameter 1**	**Parameter 2**	*p*	*p* **-value**
User control time	End round answer	−0.801	<0.001
Raw TLX	End round answer	−0.730	<0.001
Recall	End round answer	0.614	<0.001
F1	End round answer	0.553	<0.001
Precision	End round answer	0.501	<0.001
Age	End round answer	0.080	0.092
Difficulty	End round answer	−0.079	0.094
Gender	End round answer	−0.018	0.698

In addition to analyzing correlations between our main variables, we created multiple linear regression models to determine which combinations of factors led to the best predictions of users’ trust ratings. The selection criteria for the variables used in our models were based on the work of Hoff and Bashir (2015), where elements related to the development of trust are categorized according to their impact on trust prior or during the interaction with an agent. Table 4 shows the combination of factors, mean square error, and adjusted correlation coefficients for each models. Our results show that the best performance for predicting trust ratings ($R^{2}=0.915$) were achieved by combining measures related to reliance (user control time), performance (the number of shots fired, missiles destroyed and misses), task complexity and information related to the participants’ age and reported gender. These results corroborate the findings from Hoff and Bashir (2015) where elements captured during the interaction (such as performance and reliance related to “Dynamic Learned Trust” (Hoff and Bashir, 2015)] coupled to elements captured prior to the interaction (such as age and gender related to “Dispositional Trust” (Hoff and Bashir, 2015)] help us understand and be more accurate in our prediction of reported trust in the agent.

**TABLE 4:** Linear regression results when predicting participants, trust ratings from using contextual (difficulty) and behavioral measures (performance and reliance). Only the most important results are presented. A higher R2 value indicates more accurate predictions.

**Parameters**	**Mean square error**	**Adjusted** R2
User ctrl time, precision, recall, F1 difficulty, raw TLX, gender, and age	2,491.9	0.915
User ctrl time, precision, recall, F1, difficulty, and raw TLX	3,244.1	0.894
User ctrl time, precision, recall, F1, and difficulty	3,890.0	0.893
User ctrl time, precision, recall, and F1	4,717.9	0.867
Recall	17,253.2	0.793
F1	16,994.9	0.781
Precision	16,666.6	0.766
Age	14,927.5	0.686
Gender	13,634.2	0.626
Difficulty	12,517.2	0.575
Raw TLX	7,796.8	0.357
User ctrl time	1830.2	0.082

## 5 Discussion

In this paper, we have explored how agents’ predictability and reliability influence users’ perception of agents in terms of cognitive workload and trust, as well implications on task performance. As expected, we found that interacting with a nearly perfect agent (agent Epsilon) led participants to achieve higher performance while also having an overall more positive outlook of the agent. When comparing the rest of the agents, however, clear differences in users’ behaviors and perceptions were found.

With our first research question (see Section 1.1), we set out to explore how agents’ predictability impacts reliance, workload and trust. When comparing the agents with low reliability and high predictability to the agent with low reliability and low predictability (Delta), we noticed that both yielded poor overall task performance, even worse than when participants did not interact with any agent at all. These worst results were found across all performance indicators: F1, Recall and Precision. Moreover, participants had to compensate more for the agents’ inaccuracy, as is evidenced by higher user control times, greater reported workload and lower trust ratings. Nevertheless, when comparing agent Beta (low reliability, high predictability) to agent Delta (low reliability, low predictability), we found that participants performed slightly better with agent Beta, in addition to spending slightly less time correcting the agent and reporting significantly lower cognitive workloads. This suggests that when an agent’s behavior is more predictable by making errors in a systematic way, participants are able to compensate for its inaccuracy better.

When comparing agent Alpha (high reliability, high predictability) to agent Gamma (high reliability, low predictability), we found that participants achieved significantly higher performance with Alpha. They also corrected agent Alpha significantly less and reported significantly lower workload. These results further suggest that when an agents behavior is more predictable, participants could not only better compensate for the agents’ imprecision, but also adapt and work with the agent better, resulting in an overall better task performance.

Overall these findings suggest that, in the case of imperfect automation, predictability in the way an agent makes errors is important. When compared to agents with low predictability, at the same level of agents’ performance, an agent with high predictability allow users to adapt better and quicker to the agent’s behavior, which results in a higher reported trust in the agent, better task performance and reduced cognitive load.

We further hypothesized that it is possible to infer trust in an agent using information collected during human-agent interactions. To investigate this area, we first sought to determine which factors were the most important to predict participants’ perceived trust in agents. Table 3 shows correlations between trust ratings and other variables monitored in our study. While previous work hypothesized that performance is the most important predictive factor of users’ trust in agents (Hoff and Bashir, 2015), our results show that the different performance indicators used in our study (F1, Recall and Precision) are only moderately correlated with trust ratings. Moreover, our findings reveal that reliance, expressed by the amount of time users spent correcting the agents, was the metric most correlated with trust, which is in line with previous work (Dzindolet et al., 2003; Lee and See, 2004). However, we found that cognitive load (expressed with Raw Nasa TLX scores) was more strongly correlated with users’ reported trust in the agents than task performance. This finding is consistent with other work that focused on predictive decision making, where cognitive load was found to be affected by trust, reliance and the overall difficulty of the task. (Alvarado-Valencia and Barrero, 2014; Zhou et al., 2019). To further explore which combinations of factors could predict trust ratings best, we performed several multi-linear regressions. We achieved the best results (see Table 4) by using data related to users’ reliance on the agents, performance scores and the difficultly of the task. These findings suggest that it is important to consider both performance and reliance metrics in order to infer users’ trust in an agent more effectively. Moreover, we demonstrated that it is possible to predict users’ trust ratings with a very high correlation.

Our study represents a step forward toward understanding the evolution of trust in Human-Agent Interaction as it uses real-time interactions data to detect changes in users’ behaviors. However, additional tests on the variables that influence trust the most in human-agent interactions should be conducted in different contexts in order to further verify what components are the most important for the building and maintaining of the human-agent trust relationship. While in this work we only considered user control time as a measure of reliance, other behavioral measures could be included, such as the number of corrections issued by users, or the amount of time users spent monitoring the agents actions while not directly correcting them. Such measures could be used to further enhance the real-time prediction of trust in agents. The main advantages of being able to monitor this trust relationship in real-time resides in the ability to continuously monitor trusts relationships based on interactions, without the need to interrupt human operators.

## 6 Limitations

It should be noted that our study is not without limitations. We have only explored how predictability and reliability influence trust in one kind of interactive scenario in the form of a goal-oriented, collaborative aiming task. Moreover, even if initial pilots guided the design of the study, our framework is new and further work is needed to explore how our findings generalize to other real-time collaborative settings, and other populations that vary in their attitudes toward automation. In order to ensure the experiment could be completed within an hour, a number of constraints restricted the number of agents employed and the duration of the interactions. It is possible that more time spent working with the agents would help participants better calibrate their trust over time. On the other hand, interactions that are too lengthy could lead to complacency or complete distrust. In our study, however, standard deviation of trust ratings between participants was very low, which indicates that the impact of our different agents on participants was fairly consistent throughout the experiment. Furthermore, while we controlled for performance and agents behaviors, we only tested four combinations of systematic variance and bias. More agents, different levels of performance and different degrees of predictability could have been used to explore how participants’ perceptions of agents transitions from high to low trust, and less to more reliance. In addition, we realized that the performance level of our “low reliability” agents was set too low, which made it difficult for participants to perceive differences in the way they made errors. This is why the insights discussed in this paper are centered around the “high reliability” agents. We would like to note that the above limitations do not undermine the main findings of our study, but we acknowledge that additional investigations are required to understand more precisely the relationship between the different variables linked to trust in agents, as well as how other types of tasks influence this relationship. We leave these directions for future work.

## 7 Conclusion

In this study, we set out to explore the relationship between trust, agents’ predictability and agents’ reliability in a real-time collaborative scenario. To achieve this, we designed a within-groups study where participants completed a series of aiming tasks with the help of different collaborative agents. We found that, at the same level of performance, participants reported higher levels of trust in agents that were more predictable than less predictable agents. However, as the agents’ reliability decreased, participants were less trustful of the agents, regardless of their predictability. In addition, participants achieved better performance and reported lower cognitive load with systematically biased agents compared to agents with more variance, especially at a high level of agents’ performance. These findings further highlight the importance of predictability and consistency in the design of potentially error-prone agents, and how it impacts human-agent collaboration in real-time. Furthermore, our study investigated whether it was possible to infer trust ratings based on participants’ interactions. Our findings show that while performance indicators are important, in the context of real-time collaboration, participants’ reliance on agents is a better predictor of trust. These findings suggest that the development of methods that can monitor trust in automation over time is possible, and could be used by agents to better adapt to individual users. For instance, if under-reliance on an agent leads to degraded performance, “trust repair mechanisms” could be deployed to improve trust and reliance in automation and hopefully lead to improvement in overall task performance. With this work, we advance our understanding of how agent’s behavior is linked to trust, and which components influence the evolution of trust the most in real-time collaborative scenarios.

## Data Availability

The datasets used in this article are not readily available as no authorization to share the datasets from neither the Company nor the University funding this study has been given. Requests to access the datasets should be directed to sylvain.daronnat@strath.ac.uk.

## References

[B1] Ahmad M. I. Bernotat J. Lohan K. Eyssel F. (2019). Trust and Cognitive Load during Human-Robot Interaction. Virginia, USA: Association for the Advancement of Artificial Intelligence (AAAI).

[B2] Almajdalawi S. Pavlinek V. Mrlik M. Cheng Q. Sedlacik M. (2013). “Synthesis and Electrorheological Effect of Cr Doped TiO2nanorods with Nanocavities in Silicone Oil Suspensions,” in 13th International Conference on Electrorheological Fluids and Magnetorheological Suspensions (ERMR2012), Ankara, Turkey, 2–6 July 2012, 012003. 10.1088/1742-6596/412/1/012003

[B3] Alvarado-Valencia J. A. Barrero L. H. (2014). Reliance, Trust and Heuristics in Judgmental Forecasting. Comput. Hum. Behav. 36, 102–113. 10.1016/j.chb.2014.03.047

[B4] Cao A. Chintamani K. K. Pandya A. K. Ellis R. D. (2009). NASA TLX: Software for Assessing Subjective Mental Workload. Behav. Res. Methods 41, 113–117. 10.3758/brm.41.1.113 19182130

[B5] Chavaillaz A. Schwaninger A. Michel S. Sauer J. (2018). Automation in Visual Inspection Tasks: X-ray Luggage Screening Supported by a System of Direct, Indirect or Adaptable Cueing with Low and High System Reliability. Ergonomics 61, 1395–1408. 10.1080/00140139.2018.1481231 29799358

[B6] Chen J. Y. C. Lakhmani S. G. Stowers K. Selkowitz A. R. Wright J. L. Barnes M. (2018). Situation Awareness-Based Agent Transparency and Human-Autonomy Teaming Effectiveness. Theor. Issues Ergon. Sci. 19, 259–282. 10.1080/1463922x.2017.1315750

[B7] Chen T. Campbell D. Gonzalez L. F. Coppin G. (2015). “Increasing Autonomy Transparency through Capability Communication in Multiple Heterogeneous UAV Management,” in IEEE International Conference on Intelligent Robots and Systems, Hamburg, Germany, 28 Sept.-2 Oct. 2015 (IEEE). 10.1109/IROS.2015.7353707

[B8] Cohen J. (1988). “Statistical Power Analysis for the Behavioral Sciences,” in Statistical Power Analysis for the Behavioral Sciences. 2nd Edition (New York, NY: Routledge).

[B9] Correia F. Guerra C. Mascarenhas S. Melo F. S. Paiva A. (2018). “Exploring the Impact of Fault Justification in Human-Robot Trust,” in Proceedings of the 17th International Conference on Autonomous Agents and MultiAgent Systems (Richland, SC: International Foundation for Autonomous Agents and Multiagent Systems), 507–513. AAMAS ’18.

[B10] Costo S. Molfino R. (2004). “A New Robotic Unit for Onboard Airplanes Bomb Disposal,” in 35th International symposium on robotics (Paris, France: ISR (Citeseer)), 23–26.

[B11] de Visser E. J. Krueger F. McKnight P. Scheid S. Smith M. Chalk S. (2012). The World Is Not Enough: Trust in Cognitive Agents. Proc. Hum. Factors Ergon. Soc. Annu. Meet. 56, 263–267. 10.1177/1071181312561062

[B12] de Visser E. J. Monfort S. S. McKendrick R. Smith M. A. B. McKnight P. E. Krueger F. (2016). Almost Human: Anthropomorphism Increases Trust Resilience in Cognitive Agents. J. Exp. Psychol. Appl. 22, 331–349. 10.1037/xap0000092 27505048

[B13] Demir M. McNeese N. J. Cooke N. J. (2017). Team Situation Awareness within the Context of Human-Autonomy Teaming. Cogn. Syst. Res. 46, 3–12. 10.1016/j.cogsys.2016.11.003

[B14] Dzindolet M. T. Peterson S. A. Pomranky R. A. Pierce L. G. Beck H. P. (2003). The Role of Trust in Automation reliance. Int. J. Human-Computer Stud. 58, 697–718. 10.1016/s1071-5819(03)00038-7

[B15] Emmerich K. Ring P. Masuch M. (2018). “I'm Glad You Are on My Side,” in The Annual Symposium on Computer-Human Interaction in Play Extended Abstracts - CHI PLAY ’18, Melbourne VIC Australia (New York, NY: Association for Computing Machinery), 141–152. 10.1145/3242671.3242709

[B16] Entin E. E. Serfaty D. (2017). Sequential Revision of Belief, Trust Type, and the Order Effect. Hum. Factors 59, 407–419. 10.1177/0018720816678322 27941162

[B17] Fan X. McNeese M. Sun B. Hanratty T. Allender L. Yen J. (2010). Human-Agent Collaboration for Time-Stressed Multicontext Decision Making. IEEE Trans. Syst. Man. Cybern. A. 40, 306–320. 10.1109/tsmca.2009.2035302

[B18] Fan X. Oh S. McNeese M. Yen J. Cuevas H. Strater L. (2008). “The Influence of Agent Reliability on Trust in Human-Agent Collaboration,” in Proceedings of the 15th European conference on Cognitive ergonomics: the ergonomics of cool interaction, Funchal Portugal (New York, NY: ACM), 1–8.

[B19] Freedy A. DeVisser E. Weltman G. Coeyman N. (2007). “Measurement of Trust in Human-Robot Collaboration,” in Proceedings of the 2007 International Symposium on Collaborative Technologies and Systems (New York, NY: CTS), 106–114.

[B20] Gholami B. Haddad W. M. Bailey J. M. (2018). “AI in the ICU: In the Intensive Care Unit, Artificial Intelligence Can Keep Watch,”, 31–35. 10.1109/MSPEC.2018.8482421 IEEE Spectr. 55

[B21] Grodzinsky F. S. Miller K. W. Wolf M. J. (2011). Developing Artificial Agents Worthy of Trust: "Would You Buy a Used Car from This Artificial Agent?" Ethics Inf. Technol. 13, 17–27. 10.1007/s10676-010-9255-1

[B22] Hart S. G. (2006). “Nasa-task Load index (Nasa-tlx); 20 Years Later,” in Proceedings of the human factors and ergonomics society annual meeting (Los Angeles, CA): Sage publications Sage CA), 904–908. 10.1177/154193120605000909 Proc. Hum. Factors Ergon. Soc. Annu. Meet. 50

[B23] Hoc J.-M. Young M. S. Blosseville J.-M. (2009). Cooperation between Drivers and Automation: Implications for Safety. Theor. Issues Ergon. Sci. 10, 135–160. 10.1080/14639220802368856

[B24] Hoff K. A. Bashir M. (2015). Trust in Automation. Hum. Factors 57, 407–434. 10.1177/0018720814547570 25875432

[B25] Hoffman R. R. Johnson M. Bradshaw J. M. Underbrink A. (2013). Trust in Automation. IEEE Intell. Syst. 28, 84–88. 10.1109/MIS.2013.24

[B26] Honig S. Oron-Gilad T. (2018). Understanding and Resolving Failures in Human-Robot Interaction: Literature Review and Model Development. Front. Psychol. 9, 861. 10.3389/fpsyg.2018.00861 29962981 PMC6013580

[B27] Jensen T. Khan M. M. H. Albayram Y. Fahim M. A. A. Buck R. Coman E. (2020). Anticipated Emotions in Initial Trust Evaluations of a Drone System Based on Performance and Process Information. Int. J. Human-Computer Interaction 36, 316–325. 10.1080/10447318.2019.1642616

[B28] Jian J.-Y. Bisantz A. M. Drury C. G. (2000). Foundations for an Empirically Determined Scale of Trust in Automated Systems. Int. J. Cogn. Ergon. 4, 53–71. 10.1207/s15327566ijce0401_04

[B29] Karikawa D. Aoyama H. Takahashi M. Furuta K. Wakabayashi T. Kitamura M. (2013). A visualization tool of en route air traffic control tasks for describing controller's proactive management of traffic situations. Cogn. Tech. Work 15, 207–218. 10.1007/s10111-012-0222-y

[B30] Kim D.-j. Lim Y.-k. (2019). “Co-Performing Agent,” in Proceedings of the 2019 CHI Conference on Human Factors in Computing Systems - CHI ’19 (New York, New York, USA: ACM Press), 1–14. 10.1145/3290605.3300714

[B31] Klein G. Woods D. D. Bradshaw J. M. Hoffman R. R. Feltovich P. J. (2004). Ten Challenges for Making Automation a "Team Player" in Joint Human-Agent Activity. IEEE Intell. Syst. 19, 91–95. 10.1109/MIS.2004.74

[B32] Kunze A. Summerskill S. J. Marshall R. Filtness A. J. (2019). Automation Transparency: Implications of Uncertainty Communication for Human-Automation Interaction and Interfaces. Ergonomics 62, 345–360. 10.1080/00140139.2018.1547842 30501566

[B33] Lee J. D. See K. A. (2004). Trust in Automation: Designing for Appropriate reliance. hfes 46, 50–80. 10.1518/hfes.46.1.50.30392 15151155

[B34] Mercado J. E. Rupp M. A. Chen J. Y. Barnes M. J. Barber D. Procci K. (2015). Intelligent Agent Transparency in Human-Agent Teaming for Multi-UxV Management. Hum. Factors 58, 401–415. 10.1177/0018720815621206 26867556

[B35] Merritt S. M. Heimbaugh H. LaChapell J. Lee D. (2013). I Trust it, but I Don't Know Why. Hum. Factors 55, 520–534. 10.1177/0018720812465081 23829027

[B36] Merritt S. M. Lee D. Unnerstall J. L. Huber K. (2015). Are Well-Calibrated Users Effective Users? Associations between Calibration of Trust and Performance on an Automation-Aided Task. Hum. Factors 57, 34–47. 10.1177/0018720814561675 25790569

[B37] NASA (1986). Task Load index, Nasa Tlx, v1.0. NASA 1, 25–26.

[B38] Newn J. Velloso E. Allison F. Abdelrahman Y. Vetere F. (2017). “Evaluating Real-Time Gaze Representations to Infer Intentions in Competitive Turn-Based Strategy Games,” in Proceedings of the Annual Symposium on Computer-Human Interaction in Play (New York, NY, USA: Association for Computing Machinery)), 541–552. CHI PLAY ’17. 10.1145/3116595.3116624

[B39] Ogreten S. Lackey S. Nicholson D. (2010). “Recommended Roles for Uninhabited Team Members within Mixed-Initiative Combat Teams,” in 2010 International Symposium on Collaborative Technologies and Systems (IEEE). 10.1109/cts.2010.5478468

[B40] Pak R. Fink N. Price M. Bass B. Sturre L. (2012). Decision Support Aids with Anthropomorphic Characteristics Influence Trust and Performance in Younger and Older Adults. Ergonomics 55, 1059–1072. 10.1080/00140139.2012.691554 22799560

[B41] Robinette P. Howard A. M. Wagner A. R. (2017). Effect of Robot Performance on Human-Robot Trust in Time-Critical Situations. IEEE Trans. Human-mach. Syst. 47, 425–436. 10.1109/thms.2017.2648849

[B42] Rossi A. Dautenhahn K. Koay K. L. Walters M. L. (2018). The Impact of Peoples' Personal Dispositions and Personalities on Their Trust of Robots in an Emergency Scenario. Paladyn, J. Behav. Robotics 9, 137–154. 10.1515/pjbr-2018-0010

[B43] Salem M. Lakatos G. Amirabdollahian F. Dautenhahn K. (2015). “Would You Trust a (Faulty) Robot? Effects of Error, Task Type and Personality on Human-Robot Cooperation and Trust,” in 2015 10th ACM/IEEE International Conference on Human-Robot Interaction (HRI) (IEEE), 1–8.

[B44] Schaefer K. E. Chen J. Y. C. Szalma J. L. Hancock P. A. (2016). A Meta-Analysis of Factors Influencing the Development of Trust in Automation. Hum. Factors 58, 377–400. 10.1177/0018720816634228 27005902

[B45] Sgobba T. (2019). B-737 max and the Crash of the Regulatory System. J. Space Saf. Eng. 6, 299–303. 10.1016/j.jsse.2019.09.006

[B46] Sheridan T. (1989). “Trustworthiness of Command and Control Systems,” in Analysis, Design And Evaluation Of Man–Machine Systems 1988 (Elsevier), 427–431. 10.1016/B978-0-08-036226-7.50076-4

[B47] Shirado H. Christakis N. A. (2017). Locally Noisy Autonomous Agents Improve Global Human Coordination in Network Experiments. Nature 545, 370–374. 10.1038/nature22332 28516927 PMC5912653

[B48] Singh I. L. Molloy R. Parasuraman R. (1993a). Automation- Induced "Complacency": Development of the Complacency-Potential Rating Scale. Int. J. Aviation Psychol. 3, 111–122. 10.1207/s15327108ijap0302_2

[B49] Singh I. L. Molloy R. Parasuraman R. (1993b). Automation- Induced "Complacency": Development of the Complacency-Potential Rating Scale. Int. J. Aviation Psychol. 3, 111–122. 10.1207/s15327108ijap0302_2

[B50] Sordoni A. Briot J.-P. Alvarez I. Vasconcelos E. de Azevedo Irving M. Melo G. (2010). Design of a Participatory Decision Making Agent Architecture Based on Argumentation and Influence Function - Application to a Serious Game about Biodiversity Conservation. Rairo-oper. Res. 44, 269–283. 10.1051/ro/2010024

[B51] Stowers K. Kasdaglis N. Rupp M. Chen J. Barber D. Barnes M. (2017). Insights into Human-Agent Teaming. Intell. agent transparency uncertainty 499. 10.1007/978-3-319-41959-6_13

[B52] Tjøstheim T. A. Johansson B. Balkenius C. (2019). “A Computational Model of Trust-, Pupil-, and Motivation Dynamics,” in Proceedings of the 7th International Conference on Human-Agent Interaction (New York, NY, USA: Association for Computing Machinery)), 179–185. HAI ’19. 10.1145/3349537.3351896

[B53] Wang N. Pynadath D. V. Hill S. G. (2015). “The Impact of POMDP-Generated Explanations on Trust and Performance in Human-Robot Teams,” in AAMAS '16: Proceedings of the 2016 International Conference on Autonomous Agents & Multiagent Systems, Singapore (Richland, SC: International Foundation for Autonomous Agents and Multiagent Systems), 997–1005.

[B54] Wiebe E. N. Lamb A. Hardy M. Sharek D. (2014). Measuring Engagement in Video Game-Based Environments: Investigation of the User Engagement Scale. Comput. Hum. Behav. 32, 123–132. 10.1016/j.chb.2013.12.001

[B55] Williams E. (1949). Experimental Designs Balanced for the Estimation of Residual Effects of Treatments. Aust. J. Chem. 2, 149. 10.1071/ch9490149

[B56] Zhou J. Li Z. Hu H. Yu K. Chen F. Li Z. (2019). “Effects of Influence on User Trust in Predictive Decision Making,” in Extended Abstracts of the 2019 CHI Conference on Human Factors in Computing Systems (New York, NY: ACM). 10.1145/3290607.3312962

